# Is laparoscopic colorectal surgery with continuation of antiplatelet therapy safe without increasing bleeding complications?

**DOI:** 10.1007/s00595-019-01839-0

**Published:** 2019-06-22

**Authors:** Kazuhiro Taguchi, Manabu Shimomura, Hiroyuki Egi, Minoru Hattori, Shoichiro Mukai, Masatoshi Kochi, Haruki Sada, Yusuke Sumi, Ikki Nakashima, Shintaro Akabane, Koki Sato, Hideki Ohdan

**Affiliations:** 1 Department of Gastroenterological and Transplant Surgery, Graduate School of Biomedical and Health Sciences, Hiroshima University, 1-2-3 Kasumi Minami-ku, Hiroshima, Hiroshima 734-8551 Japan; 2 Institute for Clinical Research, National Hospital Organization, Kure Medical Center and Chugoku Cancer Center, 3-1 Aoyamacho, Kure, Hiroshima 737-0023 Japan; 3 Department of Surgery, Hiroshima City Asa Citizens Hospital, 2-1-1 Kabeminami, Asakita-ku, Hiroshima, 731-0293 Japan; 4 Advanced Medical Skills Training Center, Institute of Biomedical and Health Sciences, Hiroshima University, 1-2-3 Kasumi Minami-ku, Hiroshima, Hiroshima 734-8551 Japan

**Keywords:** Laparoscopic colorectal surgery, Antiplatelet therapy, Surgical outcome

## Abstract

**Purpose:**

The number of patients on antiplatelet therapy (APT) who need surgery is increasing; however, it is unclear whether APT should be continued for abdominal surgery, particularly laparoscopic colorectal surgery. We investigated the safety of continuing APT for patients undergoing laparoscopic colorectal surgery.

**Methods:**

We collected retrospective data from 529 patients who underwent laparoscopic colorectal surgery at Hiroshima University between January, 2013 and December, 2018. We analyzed information related to APT. Thirty-six pairs were matched by the propensity score method between patients on APT (APT+) and those not on APT (APT−). We compared the surgical outcomes of both groups.

**Results:**

Among 463 patients eligible for the study, 48 were on APT for cerebrovascular or cardiovascular disease, and 36 continued to take aspirin. In the case-matched comparison, the amount of intraoperative blood loss in the APT+ group was not significantly higher than that in the APT− group, and the incidences of bleeding complications, thromboembolic complications, and other complications were not significantly different between the groups.

**Conclusion:**

In a case-matched comparison, continuation of aspirin during laparoscopic colorectal surgery did not increase perioperative complications. In laparoscopic colorectal surgery, continuation of aspirin is an acceptable strategy for patients with thromboembolic risk caused by interruption of APT.

## Introduction

Antiplatelet therapy (APT) is indicated for the prevention of primary and late thrombotic complications in patients with thrombotic diseases, such as cerebrovascular or cardiovascular disease. The number of patients with thrombotic disease is increasing in line with our aging population and deliberate antithrombotic management during abdominal surgical treatment is often required. Although interruption of APT is considered perioperatively to prevent excessive hemorrhagic risk, this can result in thrombotic events in patients with thrombotic diseases [1, 2]. Biondi-Zoccai reported that the withdrawal of aspirin treatment had ominous prognostic implications for patients at moderate-to-high risk for coronary artery disease and concluded that aspirin should be discontinued only when bleeding risk clearly overwhelms that of thrombotic events [3]. However, it remains unclear which situations should be given priority to interrupt APT. Although APT reduces the perioperative risk of thrombotic complications, it may increase the risk not only of worse intraoperative blood loss, but also of postoperative bleeding complications [4]. A meta-analysis revealed that aspirin continuation was not generally associated with higher mortality rates or poorer surgery outcomes, but it was associated with a 1.5-fold increase in perioperative blood loss [5]. Another meta-analysis suggested that APT at the time of non-cardiac surgery confers minimal bleeding risk with no difference in thrombotic complications [6]. Based on the findings of these investigations of intraoperative bleeding risk from APT, the safety profile for laparoscopic colorectal surgery while continuing APT remains unclear.

Laparoscopic colorectal surgery has become broadly accepted based on large multicenter randomized controlled trials, which show less blood loss [7–11]. However, it has not been extensively investigated whether APT affects the perioperative outcomes of patients undergoing laparoscopic colorectal surgery. Although some reports describe that laparoscopic colorectal surgery is safe for patients with APT [12, 13], it is still difficult to establish the appropriate indications for the continuation of APT because surgical invasiveness in laparoscopic colorectal surgery differs among surgical procedures and is influenced by oncological status such as tumor stage. We investigated the safety of APT for laparoscopic colorectal surgery with the continuation of APT, while considering variations in the surgical procedures.

## Methods

### Patients

We collected data retrospectively from 435 consecutive patients who underwent laparoscopic colorectal surgery for clinically diagnosed adenocarcinoma, at Hiroshima University Hospital between January 2013 and December 2018. We excluded 18 patients who had multiple cancers, 3 who underwent cholecystectomy simultaneously, 18 who underwent robotic system surgery, 4 who underwent total colectomy, and 23 who were on chronic oral anticoagulation therapy. For each of the total 463 patients, we obtained the following data: sex, age, body mass index (BMI), information on comorbidity including antiplatelet therapy, American Society of Anesthesiologists (ASA) class, performance status (PS) (World Health Organization), risk classification of venous thromboembolism (VTE) based on the Caprini score [14], tumor location, lymphadenectomy, surgical curability, tumor stage, and surgical outcome such as operative time, blood loss, intraoperative blood transfusion, open conversion, morbidity, and mortality. Morbidity included re-operation within 48 h, drop in hemoglobin level (> 2.0 g/dL), postoperative surgical site and other site bleeding, venous thromboembolism (VTE), cerebral or myocardial infarction, and other complications.

### Perioperative management of antiplatelet therapy

**Fig. 1:**
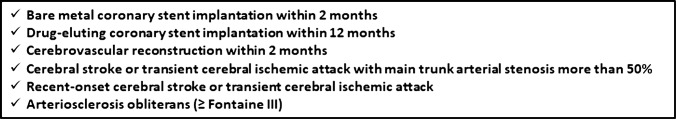
High thromboembolic risk associated with interruption of an antiplatelet agent. This list was cited from the guidelines of the Japan Gastroenterological Endoscopy Society

We identified high thromboembolic risk patients comprehensively, and basically, following the guidelines of the Japan Gastroenterological Endoscopy Society (Fig. 1) [15]. APT was generally interrupted 1 week before surgery and restarted in the early postoperative period for low thromboembolic risk patients. In high thromboembolic risk patients, APT was continued in the form of aspirin alone or heparin bridging treatment. If patients were on chronic oral anticoagulation therapy, they were managed by the interruption of anticoagulation 5–7 days before surgery, bridging anticoagulation with unfractionated heparin, and early postoperative reinstitution. In patients on both APT and oral anticoagulation therapy, the perioperative management of APT was combined with that of anticoagulation therapy. We excluded patients who were managed by heparin bridging from analysis of the safety of APT, because heparin bridging would be an independent risk factor for postoperative bleeding complications [16]. We managed VTE as outlined in our previous reports [17, 18]. Mechanical prophylaxis against VTE, including the postoperative use of elastic stockings and intermittent pneumatic compression was routine for all patients. Pharmacological prophylaxis was administered to those at clinically high risk of VTE categorized by institutional risk classification, as low molecular weight heparin: enoxaparin sodium [via subcutaneous injection, twice a day, with enoxaparin sodium (2000 IU) for 1 week].

### Statistical analysis

Summary statistics were calculated using frequencies and proportions for categorical data and medians and ranges for continuous variables. Chi square tests were used for categorical data, and Mann–Whiney *U* tests and Kruskal–Wallis tests were used for continuous data.

Since there were differences in patient backgrounds and the style of operation among patients who continued to take aspirin (APT+) and those not on APT (APT−), we used a propensity score method to balance the observed covariates related to surgical invasiveness between the groups. Among patient factors, oncological factors and surgical factors, we chose the following as covariates which might affect surgical outcomes: sex, age, BMI, ASA-class, VTE risk classification, tumor location, lymphadenectomy, surgical curability and tumor stage. One-to-one propensity matching was performed using a caliper index of 0.20. Statistical analyses were performed using JMP13 for Windows (SAS Institute). *P* values of < 0.05 were considered significant.

## Results

**Fig. 2:**
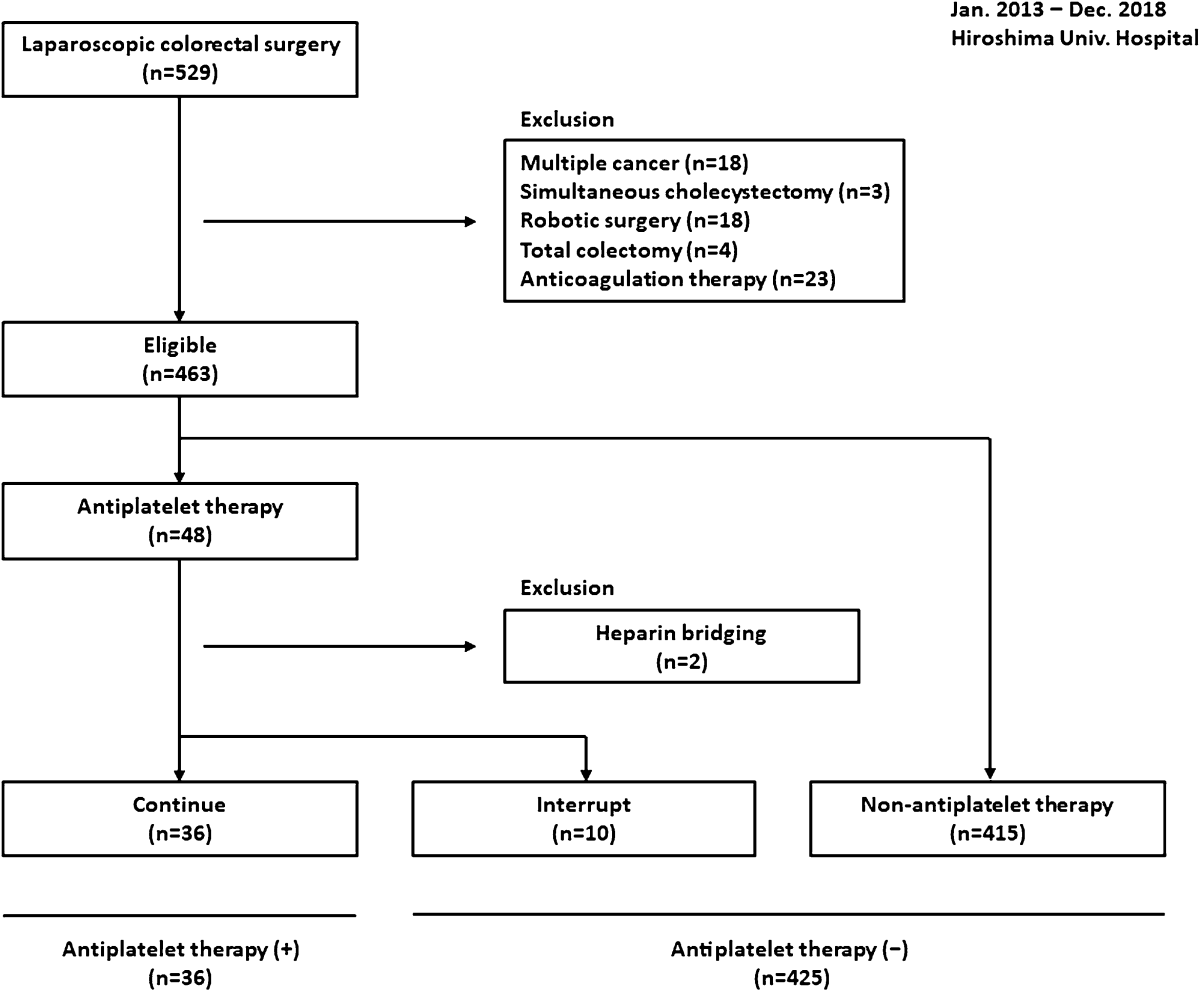
Patient flow diagram

**Fig. 3:**
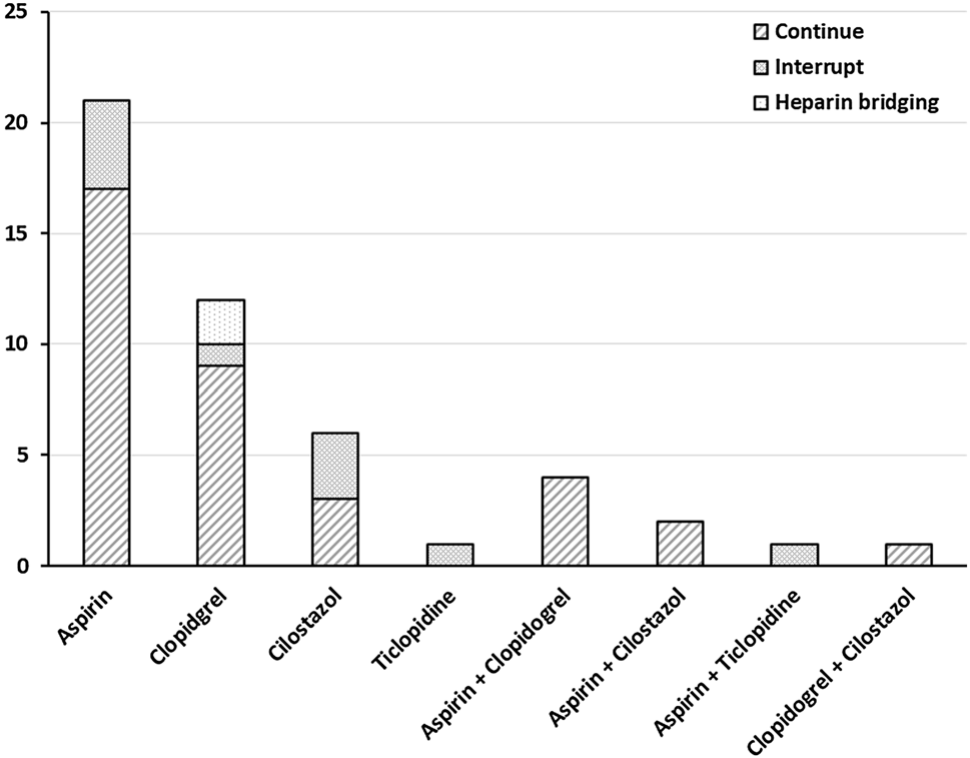
Type of antiplatelet agents. Forty-eight patients used antiplatelet agents, which were interrupted in 10 patients (20.8%), while 2 patients (4.1%) were managed by heparin bridging during the perioperative period

There were 463 patients in the final analysis, 48 of whom had been on APT for cerebrovascular or cardiovascular disease. Of those, 36 underwent laparoscopic colorectal surgery while continuing to take aspirin and 2 were managed by heparin bridging instead of continuation of APT. For the other 10 patients identified as being at low thromboembolic risk, APT was interrupted during the perioperative management (Fig. 2). The types of antiplatelet agents being used by these 48 patients were aspirin (*n* = 21), clopidogrel (*n* = 12), cilostazol (*n* = 6), ticlopidine (*n* = 1), a combination of aspirin and clopidogrel (*n* = 4), aspirin and cilostazol (*n* = 2), aspirin and ticlopidine (*n* = 1), and clopidogrel and cilostazol (*n* = 1) (Fig. 3). APT was interrupted in four patients who had been on aspirin, one who had been on clopidogrel, three who had been on cilostazol, one who had been on ticlopidine, and one who had been on a combination of aspirin and ticlopidine. Two of the patients who had been taking clopidogrel were managed by heparin bridging. Table 1 summarizes the patient characteristics and oncological status of patients who had been on APT vs. those who had not (N-APT). The proportion of men was significantly higher in the APT group (*P* = 0.0276). The median ages were 75 and 67 years in the APT group vs. the N-APT group, respectively (*P* < 0.0001). Both ASA-class and PS were poorer in the APT group (*P* < 0.0001, *P* = 0.0039, respectively). The proportion of patients with diabetes was significantly higher in the APT group (*P* = 0.0018). VTE risk classification was also significantly higher in the APT group (*P* = 0.0068). Oncological status was not significantly different between the groups. The patient characteristics and surgical outcomes of the patients who continued on APT (C-APT) vs. those with interrupted APT (I-APT) were not significantly different (Tables 2, 3, respectively). Next, we summarized the patient characteristics in the APT+ group vs. the APT− group. The median ages were 73 and 67 years, respectively (*P* < 0.0001), and the median BMI was 23.7 and 22.3, respectively (*P* = 0.0345). Both ASA-class and PS were poorer in the APT+ group (*P* < 0.0001, *P* = 0.0139, respectively). The proportion of patients with diabetes was significantly higher in the APT+ group (*P* = 0.0021). VTE risk classification was significantly higher in the APT+ group (*P* = 0.0018). For surgical factors, the distribution for the level of lymphadenectomy was significantly different in the two groups (*P* = 0.0212) (Table 4).

**Table 1:** Patient characteristics and oncological status

	Total (*n* = 463) *n* (%)	APT (*n* = 48) *n* (%)	N-APT (*n* = 415) *n* (%)	*P* value
Sex				0.0276
Male	281 (60.7)	36 (75.0)	245 (59.0)	
Female	182 (39.3)	12 (25.0)	170 (41.0)	
Age (year old) [median (range)]	67 (22–94)	75 (52–90)	67 (69–94)	< 0.0001
BMI [median (range)]	22.4 (13.7–36.0)	23.5 (15.9–32.7)	22.4 (13.7–36.0)	0.2262
ASA-class				< 0.0001
1	101 (21.8)	2 (4.2)	99 (23.9)	
2	337 (72.8)	34 (70.8)	303 (73.0))	
3	25 (5.4)	12 (25.0)	13 (3.1)	
PS				0.0039
0	407 (87.9)	36 (75.0)	371 (89.4)	
1	48 (10.4)	12 (25.0)	36 (8.7)	
2	8 (1.7)	0	8 (1.9)	
Diabetes				0.0018
Yes	388 (83.8)	16 (33.3)	59 (14.2)	
No	75 (16.2)	32 (66.7)	356 (85.8)	
VTE risk classification				0.0058
Low	67 (14.4)	1 (2.1)	66 (15.9)	
High	267 (57.7)	29 (60.4)	238 (57.4)	
Highest	129 (27.9)	18 (37.5)	111 (26.7)	
Tumor location				0.2391
Right	148 (32.0)	19 (39.6)	129 (31.1)	
Left	142 (30.7)	10 (20.8)	132 (31.8)	
Rectum	173 (37.3)	19 (39.6)	154 (37.1)	
Stage				0.1282
0	27 (5.8)	0	27 (6.5)	
I	225 (48.6)	25 (52.1)	200 (48.2)	
II	83 (18.0)	7 (14.6)	76 (18.3)	
III	101 (21.8)	12 (25.0)	89 (21.5)	
IV	27 (5.8)	4 (8.3)	23 (5.5)	

*BMI* body mass index, *ASA* American Society of Anesthesiologists, *PS* performance status, *VTE* venous thromboembolism

**Table 2:** Patient characteristics in the continuing antiplatelet therapy (C-APT) and interrupted antiplatelet therapy (I-APT) groups

	C-APT (*n* = 36) *n* (%)	I-APT (*n* = 10) *n* (%)	*P* value
Sex			0.7527
Male	27 (75.0)	7 (70.0)	
Female	9 (25.0)	5 (33.3)	
Age (year old) [median (range)]	73 (52–85)	78 (69–90)	0.2092
BMI [median (range)]	23.7 (17.1–32.7)	21.0 (15.9–28.7)	0.1874
ASA-class			0.5532
1	2 (5.6)	0	
2	25 (69.4)	8 (80.0)	
3	9 (25.0)	2 (20.0)	
PS			0.2773
0	27 (75.0)	9 (90.0)	
1	9 (25.0)	1 (10.0)	
2	0	0	
Diabetes			0.3206
Yes	13 (36.1)	2 (20.0)	
No	23 (63.9)	8 (80.0)	
VTE risk classification			0.1314
Low	0	1 (10.0)	
High	22 (61.1)	7 (70.0)	
Highest	14 (38.9)	2 (20.0)	

*BMI* body mass index, *ASA* American Society of Anesthesiologists, *PS* performance status, *VTE* venous thromboembolism

**Table 3:** Surgical outcomes of the continuing antiplatelet therapy (C-APT) and interrupted antiplatelet therapy (I-APT) groups

	C-APT (*n* = 36) *n* (%)	I-APT (*n* = 10) *n* (%)	*P* value
Operative time (min) [median (range)]	305 (69–627)	285 (158–372)	0.4243
Blood loss (ml) [median (range)]	57 (5–483)	16 (5–240)	0.0567
Intraoperative blood transfusion			–
Yes	0	0	
No	36 (100)	10 (100)	
Open conversion			0.3160
Yes	2 (5.6)	0	
No	34 (94.4)	10 (100)	
Re-operation within 48 h			–
Yes	0	0	
No	36 (100)	10 (100)	
Hb drop (> 2.0)			0.1508
Yes	4 (11.1)	0	
No	32 (88.9)	10 (100)	
Postoperative surgical sight bleeding			–
Yes	0	0	
No	36 (100)	10 (100)	
Postoperative other sight bleeding			–
Yes	0	0	
No	36 (100)	10 (100)	
VTE			0.4811
Yes	1 (2.8)	0	
No	35 (97.2)	10 (100)	
Cerebral or myocardial infarction			–
Yes	0	0	
No	36 (100)	10 (100)	

*Hb* hemoglobin, *VTE* venous thromboembolism

**Table 4:** Patient characteristics and surgical factors overall and after propensity score matching in the antiplatelet therapy (APT+) vs. no antiplatelet therapy (APT−) groups

	APT+ (*n* = 36) *n* (%)	APT−			
		Overall (*n* = 425) *n* (%)	*P* value	After matching (*n* = 36) *n* (%)	*P* value
Sex			0.0569		0.7891
Male	27 (75.0)	252 (59.3)		26 (72.2)	
Female	9 (25.0)	173 (40.7)		10 (27.8)	
Age (year old) [median (range)]	73 (52–85)	67 (22–94)	< 0.0001	78 (57–90)	0.4773
BMI [median (range)]	23.7 (17.1–32.7)	22.3 (13.7–36.0)	0.0345	23.6 (15.9–30.1)	0.7525
ASA-class			< 0.0001		0.8780
1	2 (5.6)	99 (23.3)		3 (8.3)	
2	25 (69.4)	311 (73.2)		25 (69.4)	
3	9 (25.0)	15 (3.5)		8 (22.2)	
VTE risk classification			0.0018		0.4749
Moderate	0	67 (15.8)		0	
High	22 (61.1)	245 (57.6)		19 (52.8)	
Highest	14 (38.9)	113 (26.6)		17 (47.2)	
Tumor location			0.2834		0.9494
Right	14 (38.9)	134 (31.5)		13 (36.1)	
Left	7 (19.4)	134 (31.5)		8 (22.2)	
Rectum	15 (41.7)	157 (37.0)		15 (41.7)	
Lymphadenectomy			0.0212		1.0000
D2	27 (75.0)	237 (55.8)		27 (75.0)	
D3	9 (25.0)	188 (44.2)		9 (25.0)	
Surgical curability			0.9213		0.5517
R0	34 (94.4)	401 (94.4)		35 (97.2)	
R1	0	1 (0.2)		0	
R2	2 (5.6)	23 (5.4)		1 (2.8)	
Stage			0.1713		0.8632
0	0	27 (6.3)		0	
I	21 (58.3)	203 (47.8)		24 (66.7)	
II	4 (11.1)	78 (18.3)		3 (8.3)	
III	9 (25.0)	92 (21.7)		8 (22.2)	
IV	2 (5.6)	25 (5.9)		1 (2.8)	

*BMI* body mass index, *ASA* American Society of Anesthesiologists, *VTE* venous thromboembolism

Since APT may increase the risks related to perioperative bleeding complications, we compared the surgical short outcomes between the APT+ and APT− groups. In this cohort, patient background and surgical invasiveness, including the level of lymphadenectomy, differed between the groups. We selected 36 matched pairs from among these patients by the propensity score method and found no significant difference in any patient characteristics or surgical factor between the groups after matching (Table 4). In these matched pairs, the intraoperative blood loss in the APT+ group was not significantly higher than that in the APT− group (median, 57 and 54 ml, respectively, *P* = 0.5026). There were no significant differences in bleeding complications, thromboembolic complications, and other complications between the APT+ and APT− groups (Table 5).

**Table 5:** Surgical outcomes of the antiplatelet therapy (APT+) and no antiplatelet therapy (APT−) groups

	APT+ (*n* = 36) *n* (%)	APT− (*n* = 36) *n* (%)	*P* value
Operative time (min) [median (range)]	305 (69–627)	296 (158–539)	0.8437
Blood loss (ml) [median (range)]	57 (5–483)	54 (7–359)	0.5026
Intraoperative blood transfusion			–
Yes	0	0	
No	36 (100)	36 (100)	
Open conversion			1.0000
Yes	2 (5.6)	2 (5.6)	
No	34 (94.4)	34 (94.4)	
Re-operation within 48 h			–
Yes	0	0	
No	36 (100)	36 (100)	
Hb drop (> 2.0)			0.6903
Yes	4 (11.1)	3 (8.3)	
No	32 (88.9)	33 (91.7)	
Postoperative surgical sight bleeding			0.2367
Yes	0	1 (2.8)	
No	36 (100)	35 (97.2)	
Postoperative other sight bleeding			–
Yes	0	0	
No	36 (100)	36 (100)	
VTE			0.2367
Yes	1 (2.8)	0	
No	35 (97.2)	36 (100)	
Cerebral or myocardial infarction			–
Yes	0	0	
No	36 (100)	36 (100)	
Other complications (G2)			0.5263
Yes	7 (19.4)	5 (13.9)	
No	29 (80.6)	31 (86.1)	
Other complications (> G3)			0.3231
Yes	7 (19.4)	4 (11.1)	
No	29 (80.6)	32 (88.9)	
Mortality (within 1 month)			–
Yes	0	0	
No	36 (100)	36 (100)	
Period of hospital stay (day) [median (range)]	12 (7–126)	11 (7–91)	0.1586

*Hb* hemoglobin, *VTE* venous thromboembolism

## Discussion

We investigated the safety of patients continuing APT when undergoing laparoscopic colorectal surgery. This study demonstrated that continuation of APT during laparoscopic colorectal surgery did not increase perioperative complications in a case-matched comparison.

Antithrombotic management and antibleeding management are contradictory strategies in surgical treatment. For patients on continuing antiplatelet agents, perioperative antithrombotic management during abdominal surgery is challenging due to the increased risk of perioperative bleeding and thromboembolism. When these patients undergo major abdominal surgery, interruption of APT is conventionally recommended to prevent hemorrhagic occurrence, but it has recently been considered that thromboembolic complications could be more critical than bleeding complications. Major surgery, particularly that involving the abdomen, pelvis, and lower extremities, is an important trigger of a thrombophilic state [19]. This state is supposedly further aggravated by the proinflammatory and prothrombotic effect of surgical invasion during the perioperative period. Considering this context, the optimal management of patients with APT during major abdominal surgery is still undefined. Conversely, the Japan Gastroenterological Endoscopy Society guidelines for the management of patients on antithrombotic therapy state that its continuation is indicated during a gastroenterological endoscopic procedure. We applied this guideline to patients who underwent laparoscopic colorectal surgery and high thromboembolic risk patients who continued on a single antiplatelet agent, usually aspirin.

First, to evaluate whether APT should be interrupted during laparoscopic colorectal surgery in the clinical setting, we compared surgical outcomes between patients who continued APT and those whose APT was interrupted. In this study, no thromboembolic complications, including cerebral and myocardial infarction, occurred as a result of the interruption of APT, but it was difficult to recommend interruption of APT definitively because of the small number of cases. Therefore, we focused on investigating the safety of APT through a comparison with patients not on APT.

In laparoscopic colorectal surgery, various surgical factors determine invasiveness, such as the level of lymphadenectomy. Difficulty and invasiveness differ among procedures for colorectal surgery and are basically defined by tumor location. As such, these factors affect surgical outcomes, including intraoperative blood loss and postoperative bleeding complications. While it is important to consider such variations in the analysis of surgical outcomes, the high thromboembolic risk patients in this study potentially underwent less-invasive surgery to prioritize surgical safety because such patients generally have comorbidities, resulting in poor surgical outcomes. Another study considered that the level of lymphadenectomy was reduced in elderly patients, likely to have comorbidities causing perioperative complications [20]. Those authors performed a propensity score-matching analysis using not only patient background but also surgical invasiveness, including the level of lymphadenectomy [20]. In our retrospective study, patients in the APT+ group possibly underwent less-invasive surgery than those in the APT− group; therefore, we performed a propensity score method to balance surgical invasiveness. This may be the first report to include a surgical invasiveness-matched comparison to assess antithrombotic management during laparoscopic colorectal surgery. We evaluated outcomes without the different distributions of various surgical factors to arrive at a more precise validation of APT safety.

To evaluate the safety of laparoscopic colorectal surgery while continuing APT, we also compared surgical short-term outcomes such as the amount of intraoperative blood loss, intraoperative blood transfusion, other morbidities, and mortality. It has been reported that blood loss during surgery for colon cancer influences long-term survival [21], although the permissible blood loss without the risk of other outcomes is unclear. In this comparison, the amount of blood loss was not significantly different between the APT+ and APT− groups. Moreover, in these pairs, there were no cases of critical damage caused by intraoperative bleeding and no significant differences in other complications between the groups. These results suggest that laparoscopic colorectal surgery can be performed safely on patients continuing to take APT.

This study had some limitations. First, it was a retrospective cohort study, so there was bias in the management of APT. APT was continued for patients at high thromboembolic risk as identified by guidelines, but decisions were made comprehensively about whether to continue APT. Patients who were given heparin instead of APT during the perioperative period were excluded from the analysis because it was unclear whether this alternative treatment would affect surgical outcomes. Management to prevent VTE based on the risk classification differed among patients and this might also affect bleeding and thromboembolic complications. Second, there was bias in the selection of operation style, which we resolved between the APT+ and APT− groups using the propensity score method, although left-hemicolectomy, intersphincteric resection, and Hartmann operation were not included in this analysis. Third, comparison between the APT+ and APT− groups was performed on a small sample size due to the small number of patients on APT. The incidence of bleeding complications was generally less than 5% [22]. Since the frequency of symptomatic pulmonary thromboembolism has been reported as 0.5–1.6% [19, 23, 24], it was difficult to evaluate such differences in this small sample size. In this study, there were no bleeding complications caused by the continuation of APT.

## Conclusion

In a case-matched comparison, the continuation of aspirin did not increase perioperative complications. In laparoscopic colorectal surgery, continuation of aspirin is an acceptable treatment strategy for patients at thromboembolic risk caused by an interruption of APT.

## Abbreviations

APT: Antiplatelet therapy
BMI: Body mass index
ASA: American Society of Anesthesiologists
PS: Performance status
VTE: Venous thromboembolism
PTE: Pulmonary thromboembolism
DVT: Deep-vein thrombosis
